# The Acceptability of AI-Driven Resource Signposting to Young People Using a Mental Health Peer Support App

**DOI:** 10.1007/s44206-025-00202-w

**Published:** 2025-06-04

**Authors:** Bethany Cliffe, Lucy Biddle, Jessica Gore-Rodney, Myles-Jay Linton

**Affiliations:** 1 Population Health Sciences, Bristol Medical School, University of Bristol, Bristol, UK; 2 The National Institute for Health Research Applied Research Collaboration West (NIHR ARC West) at University Hospitals Bristol and Weston NHS Foundation Trust, Bristol, UK; 3 Tellmi, London, UK; 4 School of Education, University of Bristol, Bristol, UK

**Keywords:** Artificial intelligence, Mental health, Digital interventions, Peer support, Young people

## Abstract

Incorporating artificial intelligence (AI) into mental health applications (apps) can help to personalise support, for example through signposting topic-specific resources based on content that app users interact with. However, there is limited research exploring the acceptability of AI within digital mental health for young people. The current study explored this in the context of an online peer support platform for young people. 12 young people were interviewed online using a think aloud approach; they were aged 16–23 (M 18.64, SD 2.23). Participants identified as White (*n* = 7), Chinese (*n* = 1), Mixed Race (*n* = 1), Indian (*n* = 1), Black African (*n* = 1) and Bangladeshi (*n* = 1). 10 participants identified as women, one as non-binary and one preferred not to say. Participants were users of Tellmi, a pre-moderated mental health peer support app aimed at young people. Participants were given a link to a prototype of the Tellmi app via their web browser in which it was shown how AI could generate suggestions of pre-defined resources based on the content of fictional posts. Users were encouraged to interact with it whilst thinking aloud. Three themes were developed using reflexive thematic analysis: (1) Fear of the unknown - getting to grips with artificial intelligence; (2) AI can help save time and effort by streamlining processes; and (3) The value of human connection, which included the sub-theme: AI isn’t human and shouldn’t pretend to be.

**Supplementary Information:**

The online version contains supplementary material available at 10.1007/s44206-025-00202-w.

## Introduction

The use of artificial intelligence (AI) is emerging as a way of increasing, streamlining and personalising mental healthcare (Lee et al., 2021). AI has the capability to detect patterns in large datasets that can help develop understanding, detection and management of health needs (Graham et al., 2019). Specifically, a scoping review exploring AI in mental health applications (apps) identified various functions that it can perform, including diagnosis, supportive conversations to improve wellbeing, and predicting stress, mood and risk (Milne-Ives et al., 2022). Numerous reviews have also evidenced the vast capabilities of AI within other mental healthcare spaces (D’Alfonso, 2020; Lee et al., 2021; Rebelo et al., 2023), such as processing and automating the summary of therapeutic sessions into clinical notes, wearable tech that can process bodily signals in real-time, predicting patient non-compliance and supporting treatment adherence among patients. This suggests AI can be instrumental in assessing and treating mental health difficulties via technology.

Despite the capabilities of AI there are concerns to note. In particular, issues have been raised regarding exploitation of sensitive data, displacing human connection, providing unreliable or incorrect information/advice, accessibility issues for those less digitally literate, an absence of cultural or linguistic sensitivity and perpetuated stigma or bias (Hamdoun et al., 2023). AI may perpetuate bias when it is relying on or has been trained on pre-existing bias within data, which may serve to reinforce dangerous stereotypes (i.e., confirmation bias). This can pose significant risks in both AI decision making and AI-informed human decision making (Kadiresan et al., 2022).

There are also ethical concerns around AI’s role in decision-making within young people’s mental healthcare (Valentine et al., 2023), and policies with practical guidelines on how to ensure best practices are scarce. Extensive reviews into the topic have mapped out these concerns in greater depth (Boucher et al., 2021; McCradden et al., 2023). Nevertheless, UNICEF has recommended nine requirements to be considered when implementing AI technologies for young people in relation to privacy, safety, inclusion, and transparency (UNICEF, 2021). A key recommendation from UNICEF is to work with young people so that they are included in the process of AI design and implementation. This is corroborated in literature highlighting the importance of user-centred design when incorporating AI into healthcare (Bitkina et al., 2023; Or et al., 2023).

A mixed-methods study in Germany explored young people’s attitudes towards AI-informed mental health apps, combining focus groups and an online survey (Götzl et al., 2022). They found young people have a general, broad understanding of AI. Providing that AI performed useful functions for them, young people typically perceived it positively with only 19% reporting feeling negative about its use in mental health apps. However, they were unhappy about AI using data about their personal thoughts or feelings and any clinical diagnoses they had to improve its functionality, and they did have concerns around AI’s ability to understand complex emotions. To help overcome concerns, young people wanted transparency around how AI works and is used. The need for transparency of AI is one of the UNICEF recommendations outlined above, and it has been corroborated in a qualitative workshop in the UK with young people exploring their attitudes towards AI in medicine (Visram et al., 2021). In this study it was discussed how young people’s reservations about AI may come from how it is depicted in science fiction, so transparency about how AI works may help to improve its perceived trustworthiness. Similarly, research so far has shown that young people trust more ‘human-like’ interactions from chatbots (Koulouri et al., 2022; Sia et al., 2021).

Extant research has focused largely on attitudes towards the incorporation of AI into clinical health care and the role it can play in the doctor–patient relationship (Young et al., 2021), or on the use of AI chatbots (Koulouri et al., 2022; Kretzschmar et al., 2019; Sia et al., 2021). Conversely, there is a more limited understanding of young people’s attitudes towards the use of AI within mental health apps. Following this, the current study sought to further explore how young people perceive the use of AI in mental health apps. In particular, the use of natural language processing (NLP) for signposting users to specific help sources within a directory of services was explored. NLP is a subtype of AI that uses large amounts of language-based data to learn and respond to language as a human would. This was explored in the context of Tellmi, an anonymous and pre-moderated wellbeing app where children and young people (CYP) aged 11–25 can share experiences and support other age-banded peers (please see https://www.tellmi.help/ for more information). Tellmi has around 77,700 users signed up. Within Tellmi CYP write their own posts and respond to other users’ posts on a range of topics, including wellbeing and mental health, often drawing on their own experiences to offer empathy or advice. Tellmi users also have access to a directory of 600 + resources and services covering a range of topics to enable CYP to self-manage mental health. Evidence suggests Tellmi improves mental health outcomes for young people and helps them to feel less alone (Ravaccia et al., 2022).

NLP could signpost relevant directory support resources to app users based on the content of the posts they write or interact with. Valentine (2022) identified that using AI-integrated recommendation systems in digital mental health provides users with greater opportunities to receive relevant and personalised mental health support. Additionally, these systems give individuals autonomy over their healthcare. However, the systems’ limited ability to explain the rationale for choosing such personalised recommendations, the dangers of not being explicit with the users about information storage and the potential for inaccurate recommendations are a few ethical challenges that exist when considering incorporating AI-integrated recommendation systems in digital mental health (Valentine, 2022).

It is then necessary to understand whether using NLP to facilitate signposting would be acceptable to young people in the remit of digital mental health and how it could best be implemented. In the current study, the objective was to explore the acceptability of AI-driven support signposting in the context of an online peer support platform for young people. This work contributes a deeper understanding of how young people understand and perceive AI being used in this way, which gives rise to key suggestions for how people involved in AI and mental health can ensure it is used appropriately, safely and ethically.

## Methods

### Study Design

One-to-one think-aloud interviews were held with Tellmi users online over Microsoft Teams between June and August 2023. The research team was comprised of four mental health researchers with expertise in conducting qualitative interviews of a sensitive nature with young people. Interviews in the current study were performed by someone who had enough knowledge of the app to ask appropriate probing questions, without being an ‘expert’ on the app to minimise any impact of this. The researcher had downloaded the app and explored some of its features (e.g., reading posts, filtering posts by topic, exploring the directory of resources) for approximately a month before interviews took place. This meant that they were sufficiently familiar with its content, design and user processes to enable them to discuss it with participants. Interviews lasted between 20 and 58 min (M = 34.08, SD = 9.72).

### Recruitment

Participants were young people already signed up to use the Tellmi app, and they were recruited via a notification within the app. The notification gave a brief overview of the study and linked to an online survey website that hosted the information sheet and a brief screening survey, which contained questions regarding age, gender identity, ethnicity, length of time using Tellmi, and contact details. Inclusion criteria were participants being aged 16–25, having a good grasp of the English language, and having access to a device through which they could join a Microsoft Teams call and share their screen. The demographic screening questions were to assess the diversity and representativeness of the sample. An information power approach to sample size was taken here and a sample of 12 was deemed appropriate due to the study aim being focussed, the target population being specific, there already being some well-established research and theory around the acceptability of AI in other populations and contexts, and the articulateness of participants meaning high quality interview dialogue eliciting richness and depth of data (Malterud et al., 2016). 20 app users registered interest in participating in an interview, however, three of those were too young, and five did not respond to the researchers. Consequently, interviews were conducted with 12 young people aged 16–23 (M 18.64, SD 2.23). Participants identified as women (*n* = 10) and non-binary (*n* = 1); one preferred not to say. Participants reported their ethnicities as White (n = 7), Chinese (*n* = 1), Mixed Race (*n* = 1), Indian (*n* = 1), Black African (*n* = 1) and Bangladeshi (*n* = 1). Not all participants could remember how long they had been using Tellmi for, but for those who could (*n* = 10) this ranged from ‘a few weeks’ to ‘two years’.Participants received £25 worth of Amazon vouchers to compensate for their time.

### Ethical Considerations

This study received ethical approval from the University Faculty of Health Sciences Research Ethics Committee [14081]. Participants provided verbal, recorded consent during the interview as approved by the ethics committee. Finally, participants were emailed a debrief sheet following the interview listing sources of support that they could access if required.

### Procedure

**Fig. 1:**
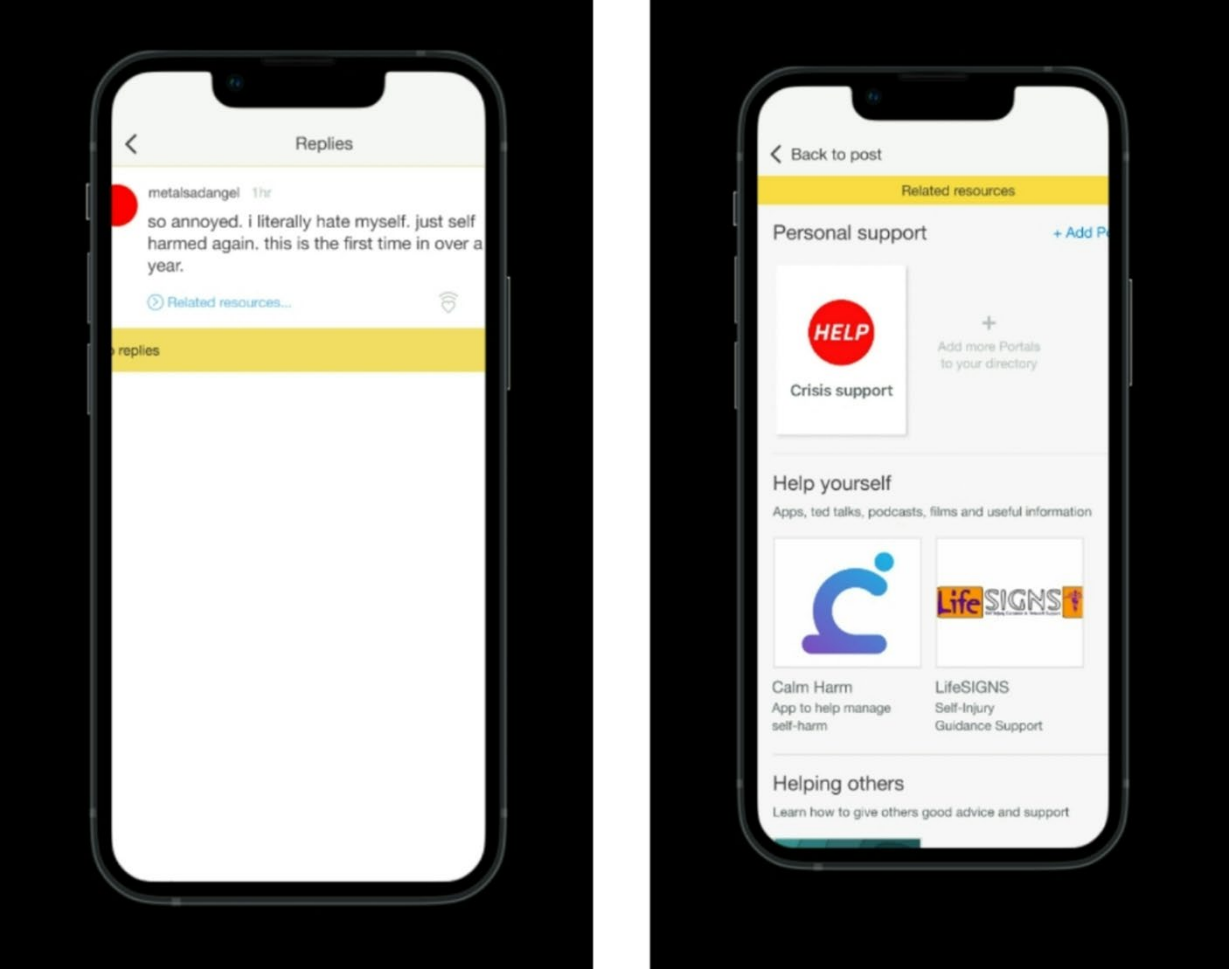
Screenshots of app prototype used during think aloud task

A think aloud process was followed, similar to Reinhart et al. (2022). Participants were first welcomed to the interview with friendly discussion to build rapport. The think aloud process was explained, as well as its purpose. Participants were informed that they may be reminded to think aloud if necessary, as it can feel unnatural at first and they may forget. Participants were then asked to access a prototype of the Tellmi app on Figma, via a link placed in the Teams chat. Through Figma, they were able to explore and interact with the app in a controlled environment to understand how the incorporation of AI would work. AI is not currently embedded within the app and so, although all participants were already users of the app, they were only exposed to the AI intervention during the one-off interview in a hypothetical prototype format for the sake of early exploratory work. In the prototype, users could interact with posts about different topics and click a ‘related resources’ button which showed them how AI could be used to suggest resources to app users relative to post content, for example if a post was about self-harm then app users would be shown related resources such as articles or user stories about self-harm, and self-help websites or apps. A Wizard-of-Oz design was used in which AI was not truly implemented in the prototype, but predefined prompts with uniform recommendations for all participants relative to each post topic were used. Participants were asked to share their screen and think aloud as they explored the app to share their thoughts on the resources suggested and the processes involved. Please see Fig. 1 for screenshots of the app prototype showing what participants saw during the think aloud task.

A topic guide was co-developed by the research team and the industry partner to address the objective and elicit participants’ opinions about AI. This included prompts for the think aloud task such as ‘what do you think of this part?’ and ‘how do you feel about what you are seeing now?’ if the participant needed reminding. After the think aloud task was completed, the interviewer asked more open questions about any areas not already covered (such as specific resources). Questions included ‘how do you feel about artificial intelligence being used in mental health apps’, ‘to what extent do you trust AI to be used in this setting’, and ‘are there any concerns or benefits you can think of for AI being used in mental health apps’. Participants typically shared their general thoughts about AI throughout the think aloud task, but to allow them the space to fully engage with the exercise the interviewer waited until after the task was exhausted to explore these in depth. Consequently, following the think aloud section the interviewer asked questions covering perceptions of AI in mental health apps compared to other platforms and how a mental health app incorporating AI would change participants’ perceptions or use of it. These questions were led by opinions each participant had already shared. Please see Online Material 1 for the topic guide.

A distress protocol was developed that could be followed if a participant became upset during the interview (Whitney & Evered, 2022). This included the input of a clinical advisor who agreed to be consulted if there was concern about the wellbeing of a participant. Fortunately, this was not needed at any point.

### Data Analysis

The interviews were all transcribed by a third party company and were analysed by the research team using reflexive thematic analysis (Braun & Clarke, 2006, 2019). Initially, BC read all transcripts to become familiar with them. Next, BC, ML and LB independently coded a transcript then met to discuss codes and possible data interpretations. Microsoft Word was used to code the data. BC then proceeded to code all transcripts, using a coding table to extract and organise codes and their corresponding data. Codes were then written up on A1 paper to identify possible themes. The themes were compared against the coding table to check whether they were a good representation of the data. The themes were discussed with the wider research team and were edited until the team felt that they adequately captured the data.

## Results

Three themes were developed: (1) Fear of the unknown - getting to grips with artificial intelligence; (2) AI can help save time and effort by streamlining processes; and (3) The value of human connection, which included the sub-theme: AI isn’t human and shouldn’t pretend to be.

### Theme 1: Fear of the Unknown - Getting to Grips with Artificial Intelligence

For many of the participants in this study, AI was described as “the unknown” [P10] which elicited “uncertainty because we don’t really understand it very well” [P11]. Despite being familiar with the concept and understanding that AI is often used in platforms such as YouTube or Snapchat, participants struggled to describe it or explain it. It seemed as though their understanding of AI was somewhat shaped by its portrayal in the news and in films, particularly negative portrayals, with participants often referring to AI as a ‘robot’. For example, misinformation about AI within the news was accredited with mistrust:

I think most people’s mistrust about AI and other technologies will be because of obviously misinformation and all these things, fake news [P5]

Similarly, perceptions of AI were sometimes through the lens of depictions in science fiction films:

I have mixed feelings about AI probably because I saw the Mission Impossible movie. I swear to you, it relates. There was this AI system called the Entity and oh my God, it’s insane. Again, it can cause a lot of help, but it can cause a heck tonne of damage as well [P11]

AI being unknown led participants to discuss their fear around its limits, such as “concerns with AI is how infinite it is” [P6], and it being “unlimited in a bad way in some aspects” [P12]. This caused anxiety around what exactly AI is capable of beyond signposting resources:

I am a bit worried about what the artificial intelligence can otherwise do […]. How much power it has, and what else it can read, and things […] I just don’t know what it can do, and whether it can track you, or anything [P1]

These concerns extended to fears around how AI will likely develop and become more intelligent over time, perhaps even overtaking human intelligence, with participants discussing that it might in fact be developing *too* quickly:

Because like if we’re thinking 20 years ago there was like not as much technology and it was all just kind of developing and everyone was like, ‘This could happen, but it might not.’ And now that it is happening it’s growing quicker than like it should be, right? (P3)

Participants also had more specific concerns around the possibilities of AI malfunctioning and issues of data protection and hacking. It seemed as though the intentions behind the AI were important in determining its acceptability, some participants noted that “There’s a lot of people who want to do this for bad reasons and also for AI you don’t actually know who is controlling it or if it’s actually in control for itself” [P3].

Conversely, AI that is designed to help – as in the context of this research - seemed to resolve participants’ doubts:

I guess it’s trying to make the site better [P1]

Similarly, knowing that there is no ulterior motive behind Tellmi incorporating AI into the app was reassuring:

I think that since it’s specifically being used to suggest resources, and not, like, to make you use the app for longer, because that’s normally what they’re trying to do, it doesn’t feel sinister. It doesn’t feel like it’s trying to make you use this app more, and see more ads, because obviously it doesn’t have ads, it’s just giving you recommendations. [P4]

Given the fear of the unknown and the importance of knowing AI’s intentions, most participants emphasised the need for transparency around its use. This included wanting to know what it is being used for, what is happening to people’s data, and who developed and is in control of the AI. It was also noted that this information needed to be presented clearly and in ways that everyone would be able to understand. This transparency was cited by participants as necessary to trust platforms that use AI:

I think just making sure that people are happy with it being used. Making sure that people have information about how it’s being used because a lot of people are scared of the unknown and having heard the things on the news about AI and that. So it’d be helpful to educate them on how their information’s being used. [P10]

### Theme 2: AI Can Help Save Time and Effort by Streamlining Processes

Despite the concerns and uncertainties around AI generally, the use of AI to suggest related resources to app users based on the content of a post was perceived positively by all participants. They believed that, in this specific context, AI could be a useful tool that could perform the function quicker and easier than a human being:

So, like in general, [AI] is scary, but in terms of this kind of thing […] it’s not so complex […]. I believe in things like this it’s extremely helpful because it’s faster, I mean it works faster than someone who has to like read the post and analyse though it and everything. [P6]

It was discussed how participants felt that AI suggesting related resources could be particularly helpful for “people (who) might not have the headspace to scroll through a lot of resources and find ones that are relevant to them” [P10]. Participants shared how it can be overwhelming and difficult trying to find resources themselves, so having AI “narrow it down” [P3] would be helpful.

Participants also felt that AI suggesting resources from a post’s content could be particularly helpful for people who are not quite sure what they are feeling, for example someone may be having suicidal thoughts but has not yet identified them as such:

I do think that it’s kind of good that if someone … like, the best example I can think of is if someone is suicidal, but doesn’t realise that that’s what they’re feeling, if they make a post talking about that, and it picks up on it, without them using the exact words, it would help people figure out what they’re feeling. If they have trouble identifying it themselves, if they make a post just explaining what they think they know about what they’re feeling, and then it comes up with resources related to suicide. [P4]

Further to resources being helpful for themselves, participants also believed that it would be helpful for making support more accessible to other users who may relate to the post:

I think definitely it’s a good thing because it does offer people resources and options, not only them but whoever relates to the post basically. [P6]

They also believed it might give app users ideas for helping a friend who may be going through something similar:

If you make a post and you’re worried about someone, if you have a friend and they’ve been self-harming and you don’t know what to do it’ll give you resources related to it, and it’ll give you maybe an idea of how they feel. [P2]

There seemed to be less concern around AI performing a task such as this, as participants believed that the ‘worst case scenario’ would be that people are still signposted to support but which may be less relevant. All participants therefore seemed reassured that AI ‘getting it wrong’ would not be harmful:

I think that it would be bound to get it wrong sometimes, but it’s still … like, I think that it could help a lot more than it would [cause harm] … it accidentally giving you the wrong resources isn’t going to be a massive issue, because it’s still a resource. […] The worst-case scenario is that you accidentally get given the wrong … like, you make a post about anxiety, and the AI interprets it as being about depression, and the worst case is that you’ve gotten help for the wrong thing. [P4]

### Theme 3: The Value of Human Connection

Conversations around AI being used in the app were often underpinned by participants expressing a preference for human connection. They described the value of the support and sense of community that they receive from peers within the app, and how this was a greater priority than the role that AI can play.

I do think [AI] can help in some ways, but, again, it means a lot more for people to connect with human beings than AI [P11]

Participants emphasised the value in having people understand their feelings and experiences, providing support and helping them to not feel so alone:

It’s a good place. I was honestly surprised that I got such positive responses and I got them quite quickly as well. I usually check every day at least, every few hours, and it really is very encouraging and heartwarming to see not just the help that you get, not just the responses that you’re getting, but also all the responses that everyone else is getting as well. So you’re all getting that bit of encouragement and support from the online community. [P11]

This experience seemed to overshadow the benefits that AI signposting support could have in a mental health app, with participants focusing more on the impact that being part of a peer support community can have. This hints at a broader view that AI is limited in the extent that it can be helpful as it is unable to deliver the most desired type of help that comes from human connection:

I think the biggest thing that is important, especially for young people, is they just want somebody to understand […] We all naturally go towards wanting to be accepted and connected and understood by somebody and to have that taken away and replaced with a robot, personally I think it’s a bit strange [laughs]. Yeah, I just don’t think that can be replaced because it’s something so real and true to being a human and to have that taken away, I don’t think it would be as good and as valuable as a human being. [P12]

### Sub-Theme: AI Isn’t Human and Shouldn’t Pretend to Be

Amongst discussions of the value of human connection was the idea that AI cannot replicate that experience, this was underscored by participants using language more suited to humans than AI to express their concerns, particularly around understanding and comprehending emotions. In this way, respondents often compared AI to humans, highlighting that AI cannot exhibit empathy or emotion. For example, the below participant expresses concern around AI being tasked with analysing data relating to complex emotions due to being a non-human entity that is not capable of emotion:

I don’t think AI would be able to understand like complex emotions […] I don’t think AI would be able to understand it from a human’s perspective just because they are not human in a way. [P3]

Participants highlighting that AI lacks human qualities was accompanied by relief in the present, but fears for a future where “[AI] will become entirely too human” [P11]. There was a clear understanding amongst participants that AI should not be humanised or attempt to mimic human experiences. This corroborates the view outlined above, that the benefits of AI are limited as it cannot provide the preferred support, i.e., human connection:

So I think from like when I was younger I got introduced to AI when it was almost newish and to me I think in a way it scared me because it’s not actually a human and by thinking that it could become human almost like it’s like you’re giving a life to something that shouldn’t have a life in a way. Because I feel like if it’s able to comprehend or like understand human emotions it should also be able to feel the same emotions. [P3]

Following this, participants were typically comfortable with AI being used to suggest related resources, but not to perform functions such as responding to posts or messaging app users:

So if it was just like helping with resources then that would be okay. But if you were expecting people to reply to you, like actual people to reply to you, it’s a bit daunting when like a robot is like, ‘Oh yeah, I’ve experienced this and I know how it feels’, but they don’t because they’re AI I guess. [P3]

Again, this was rooted in concerns around AI attempting to appear human:

I don’t like how it can write replies and seem so human, but using it to find resources and it not being like a human, I think that’s good. [P10]

Whilst participants seemed comfortable with AI being used to suggest resources, AI being a non-human entity did lead some participants to wonder if it may struggle to sufficiently “analyse” posts. For example, vague descriptions of emotions or missing out keywords in posts may mean that AI is not able to perform this function successfully. Conversely, they felt that a human would be able to interpret such nuance:

See, it would be hard for certain things because some people won’t directly say like this stuff. You know, key words that are in here like suicide lines, anxiety, self-harm. Like people might not specifically say this so then I think it would get a bit tricky to generate stuff […] See, that’s where AI gets you. Like real people they might be able to select stuff out. I don’t know. I feel like it will work if people keep direct words in, but obviously not everyone is going to do that [P2]

This may be particularly true for posts from neurodivergent users or users from a diversity of cultural backgrounds, who may express themselves differently.

I guess in the UK there’s different cultures and also obviously UK words and people express themselves in different ways I feel […] obviously the key words like ‘no one cares’ or ‘no one’s listening’ or ‘dying’, but maybe people might use words like ‘I want to fly away’ or ‘I want to end it all’. [P5]

Similarly, if someone is struggling with a panic attack or intense emotions, they may not be able to write a clear, concise post that the AI can pick up on. It may also be that a post contains multiple topics, and participants wondered if this may confuse the AI.

I do think that, like, some people, especially if they’re in the middle of … like, if they’re really panicked, in the moment, their post might not be the most, like, concise, or specific thing. So where they’re listing out their thoughts, it might be more, ‘I just need help,’ and it might not end up working too well for that. [P4]

## Discussion

### Principal Findings

Previous research has focused on the use of AI in clinical healthcare, but limited work had explored what young people think of AI in the context of mental health apps or digital peer support. This qualitative study explored young people’s perceptions of AI signposting resources based on content of a post in a peer support app. We found that, despite being familiar with the concept of AI, young people had limited knowledge around AI and how it works. Whilst we recognise a participant’s perception is a personal belief, there seemed to be possible technological misunderstanding in places. We noted several occasions where the language participants used to describe the function of AI did not match the specific analytic tasks AI models have currently been designed to complete. They also had concerns around its use due to its limits being largely unknown and it being portrayed negatively in media. They felt that it was appropriate to use AI for signposting resources as automating this process makes it quicker and easier, and risk of unintended harm is low. However, they were uncomfortable with it performing more ‘human’ functions. Similarly, young people in this study really valued the understanding, empathy, connection and sense of community they get from digital peer support, which they favoured over the role that AI can play and they were clear that AI should not be used as a surrogate to try to perform these functions. The current study has built on previous research into AI in clinical healthcare by evidencing the attitudes towards AI exhibited by young people seeking digital peer support for their wellbeing.

### Comparison with Previous Work

In the current study, participants were fearful of AI appearing too human and believed that is should not and could not replicate the human experience. This was largely due to their perception that AI lacks human qualities like empathy and understanding complex emotions, meaning it cannot provide the personal connection that they get from peer support. This has been found elsewhere (Götzl et al., 2022), however, it contrasts to other research suggesting that young people prefer more ‘human-like’ interactions with AI (Koulouri et al., 2022; Sia et al., 2021) and that young people feel chatbots can exhibit empathy and provide comfort, albeit not to the extent that a human can (Bae Brandtzæg et al., 2021). This highlights different perspectives regarding the preferences for and perceived capabilities of AI in appearing more ‘human’ or understanding emotions. Participants in the current study were users of a digital peer support app who all seemed to value the experience of human connection and were concerned about AI potentially substituting this in the future. This may help to explain why young people here were more sceptical of AI compared to those in other studies and highlights digital peer support as a niche that AI may not be able to complement. It has been suggested that allowing young people to customise what AI can do and what data it can access is important to help develop trust in it (Götzl et al., 2022). This is important as trust can predict the intention to use these technologies (Gbollie et al., 2023). Customisation of the technology has been cited as an important user need in a previous paper reporting on a workshop exploring considerations for how digital peer support can be most beneficial for young people experiencing significant distress (Cliffe et al., 2023).

There were also concerns around AI being unable to detect nuances in language relative to neurodiversity or cultural differences. A key requirement outlined in ethics guidelines for trustworthy AI is that they should foster diversity and be accessible to all to avoid marginalising groups (High-Level Expert Group on AI, 2019). Moving forward, it is essential for AI systems to be trained on a breadth of data that captures the variety of the human experience to reduce the possibilities of certain individuals being excluded or not fully benefitting from AI implementation. This is further corroborated by the need to ensure that digital mental health tools do not deepen health inequalities by restricting access or benefits based on demographics (Skorburg & Yam, 2022).

It seemed as though young people’s understanding of AI in the current study was informed by depictions in films and the news or its use in other platforms such as Snapchat, similar to Visram et al. (2021). Whilst this seems to give young people an awareness of AI, participants struggled to explain AI and suggested that they are not sure how it works or what it does. This caused anxiety, fear and uncertainty around AI in general. There is a large body of evidence suggesting the importance of explainable AI (e.g., 17–19), where transparency around its use and its processes are key. Transparency around AI has also been cited as important in evaluations of its acceptability to young people (Kretzschmar et al., 2019; Sia et al., 2021; Visram et al., 2021), and it is one of the main recommendations from UNICEF for implementing AI with young people (UNICEF, 2021) and in the ethics guidelines for trustworthy AI (High-Level Expert Group on AI, 2019). This underscores the importance of ensuring young people understand what AI is and what it is doing in services that they access. If similar research were to be conducted in a group format with young people, this may uncover further societal or cultural influences in how they understand AI when discussing it with peers.

Despite fears and uncertainties around AI, participants the current study typically found AI to be acceptable in the context of signposting resources. They believed that if it were to suggest a resource that was not wholly relevant, this would not cause much harm, if any. AI suggesting inaccurate recommendations is one ethical concern surrounding its use in mental health apps (Valentine, 2022). Whilst participants in this study were not concerned about this, it is still important to be mindful of the possibility for AI to malfunction and the consequences that this may have. If AI were to provide an inaccurate recommendation, it could leave app users feeling misunderstood, or reduce their confidence in and subsequent engagement with the app. For example, if AI incorrectly interpreted a user’s post to be about suicide and suggested resources as such, this could be unsettling for the user to be confronted with this. Equally, if it were to interpret this correctly but the user was yet to understand their own suicidality or define it as such, this may provoke a difficult realisation. Monitoring its performance and the impact of this is important to ensure that it is being used safely and appropriately, with minimal risk of harm. This is in accordance with the ethics guidelines on trustworthy AI, in which a key requirement is technical robustness including contingency plans if something malfunctions to help prevent unintended harm (High-Level Expert Group on AI, 2019). Research investigating young people’s exposure to AI within similar tools over a longer period may uncover more about the intricacies of how AI can work in practice.

### Strengths and Limitations

This study provides valuable insight into young people’s perceptions of AI and its use in digital peer support as a signposting tool, which was previously an under-researched area. A strength of this study was allowing participants to engage with a prototype of the app to demonstrate how integrating AI would look and feel within the app. This helped contextualise the discussion for participants, which may have enabled a more informed reflection on its use. However, as AI was not truly integrated into the prototype, this may have influenced participants’ perception, particularly any who were more familiar with AI systems that may generate varying responses at each interaction, for example.

We do believe the think aloud approach facilitated participants’ reflection by allowing them time and space to develop and share their insights. Whilst some participants struggled with the concept at first and required prompting, all became comfortable with thinking aloud and conversations were subsequently guided by their reflections.

As participants were mostly White women, a limitation of this study is the lack of diversity within the sample. It would have been beneficial to recruit participants with different backgrounds and identities so that their perspectives could have been represented here. Recruiting a larger sample would help with ensuring greater diversity of participants. The participants in the current study were also all recruited from within the Tellmi app, meaning they were all actively seeking digital peer support which may have influenced their perception of AI. This means that other groups of young people, such as those who do not engage with digital mental health support, may have different perspectives that are not captured here. Similarly, we have no data on participants’ mental health; it may be that the acceptability AI varies according to an individual’s level of need.

This study offers valuable insight into the perception of AI in mental health apps, and participants were able to draw inferences from their experiences with AI outside of this context. However, this research being based around one app in particular may mean that some findings are limited to this context. For example, the finding here that young people dislike AI performing/mimicking human-like functions contrasts with previous research which indicates the opposite – that young people prefer interactions with AI which are more authentically human-like. This discrepancy could be explained by the current context being peer support that has human-networking at its core.

The interviews were held online only and observing participants exploring the prototype in person may have been beneficial, however research suggests that data captured in online interviews with adolescents is comparable to ‘live’ interviews (Shapka et al., 2016). Conducting interviews online may also increase the accessibility for people who do not feel comfortable or are not able to participate in person, although requiring a laptop to participate may have meant some who wished to take part were unable to.

Finally, whilst the discussions were rooted in the app prototype, the findings are nonetheless limited to hypothetical impressions. It may be that the ‘real-world’ experience of AI signposting support may be perceived differently in the moment.

### Implications

This study found that AI-informed signposting of wellbeing resources is acceptable to young people, with the caveat that transparency around its use is key. This included transparency around what data is being used and for what purpose, so app users have clarity around what is happening with their information. If app developers were to adopt transparency when implementing AI, this would ensure peace of mind that nothing is happening to young people’s data without their knowledge and consent. This recommendation is in line with the recent EU AI act (2023), which states that an AI-based system has an obligation to inform users if AI has been involved in making any decisions or suggestions. However, the UK does not yet have such legislation in place, meaning there is a gap in the transparency that young people want and what companies are obligated to provide. In-app notifications were suggested as a way of information about AI use. As young people here had limited knowledge around how AI works and what it can do, it is clear that digital tools should present information about AI in ways that are accessible and easy to understand. App developers should work with young people to determine ways of ensuring information about AI can be presented in a way that they can understand. Simultaneously, public education should equip learners with the knowledge required to interact confidently with the technology surrounding them. This could include educating around how AI works and what its capabilities and limits are. It may be that most public coverage currently focuses on ‘what’ AI can do rather than ‘how’ AI does what it does. This is in line with recent calls for young people to receive school-based education around AI, including how it is built, its risks and the opportunities it provides (The Chartered Institute of IT, 2023). Given the accelerating pace of change, it would be beneficial to think of public education as an ongoing process where knowledge is refreshed on a recurring basis, both inside and outside of formal educational settings. Further research should explore the merits of tailoring public education dependent on intergenerational needs. For example, the key messages to convey to children interacting with technologies for the first time may differ greatly from the most pressing points to communicate to older adults who are updating their understanding of technological realities.

Given the value placed on a sense of community by participants, these findings also evidence the benefits that peer support can have for young people. It seemed that not feeling alone, being supported and developing personal connections were particularly meaningful. This is something to consider for those who support young people clinically or informally.

There are mixed findings regarding the acceptability of AI being ‘human’ like, with participants here being fearful of that. Further research is required to untangle this and to explore what may determine whether a young person responds more or less positively to AI interacting in more human ways. Understanding what is acceptable to who will be useful for providing support that is most appropriate to different groups of people. Similarly, with young people here wanting AI to be limited to non-human-like support, it is important to explore whether its use in apps such as Tellmi would be restricted to less favoured support like signposting resources such as this (Biddle et al., 2020; Cohen et al., 2022).

Participants had concerns around whether AI would be able to understand how emotions are expressed across different cultures, which hints at the issue of bias in how AI is developed and performs. If AI is trained on data that is not representative of the breadth of cultural expression, confirmation bias may occur whereby AI is not able to develop new patterns or understandings. Future research should explore the capabilities of AI with regards to cultural sensitivity to help us understand how to mitigate against bias such as this.

## Conclusions

Young people are aware of AI but may not fully understand it, which causes uncertainty and fear around its use. Whilst it is more acceptable for signposting resources, participants in this study were uncomfortable with it providing more ‘human’ like support. This was due to its perceived lack of empathy and inability to understand complex emotions. Finally, value that young people using digital peer support get from community and connections seems to overshadow the possible benefits of AI in this context.

## Electronic Supplementary Material

Below is the link to the electronic supplementary material.

Supplementary Material 1

Supplementary Material 2

## Data Availability

Due to the sensitive nature of the data and the possibility of identifying participants, data is not available. Consent was not obtained from participants to share data outside of the immediate research team.
