# Supplementary material for: The Acceptability of AI-Driven Resource Signposting to Young People Using a Mental Health Peer Support App
Source: Digit Soc. 2025 Jun 4;4(2):45. doi: 10.1007/s44206-025-00202-w (PMC12137474; doi:10.1007/s44206-025-00202-w)
Supplement: Supplementary file 1 — Supplementary Material 1 [file 44206_2025_202_MOESM1_ESM.docx]

**Article title:** The acceptability of AI-driven resource signposting to young people using a mental health peer support app.

**Journal name:** Digital Society

**Author names; affiliation and e-mail address of the corresponding author:**

Bethany Cliffe1,2, Lucy Biddle1,2*, Jessica Gore-Rodney3,

Myles Jay-Linton1,4,

1 Population Health Sciences, Bristol Medical School, University of Bristol, Bristol, United Kingdom

2 The National Institute for Health Research Applied Research Collaboration West (NIHR ARC West) at University Hospitals Bristol and Weston NHS Foundation Trust, Bristol, UK

3 Tellmi, London, United Kingdom

4 School of Education, University of Bristol, Bristol, United Kingdom

* Corresponding author

Email: lucy.biddle@bristol.ac.uk

**Topic guide**

1. How do you understand artificial intelligence?
   1. What do you know about it?
   2. What do you think it can do?
   3. Where does this understanding come from, i.e., where have you learnt about AI?
2. What do you think of artificial intelligence?
   1. Are there any concerns you have about artificial intelligence?
   2. Are there any benefits you can think of for artificial intelligence?
   3. What has influenced your opinion about AI?
3. How do you feel about artificial intelligence being used in mental health apps?
   1. To what extent do you trust AI to be used in this setting?
   2. How does AI being used in mental health apps compare to other settings?
   3. What would you think about the app if they started using AI?
      1. Would your opinion change and, if so, in what way?
4. Is there anything that would make you feel more comfortable with artificial intelligence being used in apps?
   1. Is there anything you would want to know about it?
      1. How could this best be communicated?
5. With artificial intelligence working by ‘learning’ from the data collected from within the app, how well do you think it can recommend resources that meet your needs?
   1. Are there any limits you can think of to AI learning from the data within the app?
6. Is there anything else you’d like to say that we haven’t covered?
