# Supplementary material for: The Acceptability of AI-Driven Resource Signposting to Young People Using a Mental Health Peer Support App
Source: Digit Soc. 2025 Jun 4;4(2):45. doi: 10.1007/s44206-025-00202-w (PMC12137474; doi:10.1007/s44206-025-00202-w)
Supplement: Supplementary file 2 — Supplementary Material 2 [file 44206_2025_202_MOESM2_ESM.docx]

After participants have fully explored the prototype, they are asked general questions about their attitudes towards AI and its use in the app

Interview ends and participants are emailed a debrief and thank you voucher

Participants join the interview and access a link to the Figma prototype that shows how AI could hypothetically be integrated into the app. They interact with the prototype whilst thinking aloud

Researcher contacts the participants and invites them to take part in an online interview over Microsoft Teams

Recruitment of existing Tellmi users via a notification in the app linking to an online registration of interest form
